# Disease management with ARIMA model in time series

**DOI:** 10.1590/S1679-45082013000100024

**Published:** 2013

**Authors:** Renato Cesar Sato

**Affiliations:** 1 Universidade Federal de São Paulo, São José dos Campos, SP, Brazil

**Keywords:** Time series studies, Disease management/trends, Health services administration, Intervention studies

## Abstract

The evaluation of infectious and noninfectious disease management can be done through the use of a time series analysis. In this study, we expect to measure the results and prevent intervention effects on the disease. Clinical studies have benefited from the use of these techniques, particularly for the wide applicability of the ARIMA model. This study briefly presents the process of using the ARIMA model. This analytical tool offers a great contribution for researchers and healthcare managers in the evaluation of healthcare interventions in specific populations.

## RESUMO

A avaliação do gerenciamento de doenças infecciosas e não infecciosas pode ser realizada por meio da análise de séries temporais. Com isso, espera-se que sejam medidos os resultados e previstos os efeitos da intervenção sobre a doença. Os estudos clínicos têm se beneficiado do uso dessas técnicas, visto a grande aplicabilidade do modelo ARIMA. Esse texto apresenta de maneira resumida o processo de uso do modelo ARIMA. O uso dessa ferramenta analítica oferece grande contribuição para os investigadores e gestores de saúde na avaliação das intervenções de saúde em dadas populações.

## INTRODUCTION

Disease management constitutes the ability to decrease costs of interventions within a specific population. In this type of study, the inexistence of a control group may lead to a series of bias and practical difficulties^(1)^. The approach using time series analysis is an alternative in the evaluation of disease management programs. When a time series is analyzed, the variable observed depends on its previous period, presenting a dependable series. This feature assists investigators in identifying, explaining, and predicting the effects of management programs performed throughout time. Depending on the program, inclusion of patients may not be instantaneous varying with each case. Therefore, a program that includes participants followed-up for 3 to 6 months can perceive the first results only several months or years later^(2)^. Because of the importance of “time” for disease management studies, this review presents an analysis of the autoregressive integrated moving average (ARIMA) model. This model is the most commonly used by time series health researchers^(3–6)^. Time series models have greater ability of prediction and wide applicability than nontemporal techniques^(7)^. Diffusing database use and data inclusion (eg, by using electronic medical records) creates an adequate environment for this methodology.

Some examples of the ARIMA model use include prediction of the number of beds occupied during the epidemic of severe acute respiratory syndrome (SARS) at a hospital in Singapore. Such model estimations enabled the hospital staff to predict 3 days ahead of time the number of beds that would be required during the epidemic. This study also commented on the viability of the ARIMA model for hospital bed planning and for other critical resources during epidemics of infectious diseases^(8)^. Another study conducted in China^(9)^ suggested the need for an adequate model to forecast, based on historical data, cases of hemorrhagic fever with kidney syndrome. Currently, China has 90% of cases of this disease reported globally, and the use of ARIMA models enables them to create better management and short-term predictions of the disease^(9)^.

The ARIMA model is also used as an efficient tool to plan resources such as beds and teams for the emergency department^(10,11)^. Another applicability of the ARIMA model is to predict and study antimicrobial resistance^(12–14)^.

## ARIMA MODELS

The ARIMA model was developed in the 1970s by George Box and Gwilym Jenkins as an attempt^(9)^ to describe changes on the time series using a mathematical approach. In some cases, the names ARIMA and Box-Jenkins are mentioned as synonyms. This model is based on an adjustment of observed values, and its goal is to reduce as close to zero as possible the difference between the values produced in the model and the observed ones. Quite possibly, this model can describe behaviors of stationary and nonstationary series, giving versatility to situational variances. Series are stationary when their mean and variance are constant throughout time, and when the value of covariance depends only on a gap between two time periods. Random shocks occur in nonstationary series increasing mean displacement and variance, violating the stationary condition of the series^(2)^. Some important observations in the creation of an explicative model are the need for at least 50 observations. For disease management programs, at least 4 years of data are required until the first month of intervention. Therefore, the model has the ability to place eventual patterns that could interfere in the arrangement of parameters^(2)^.

**Figure 1:**
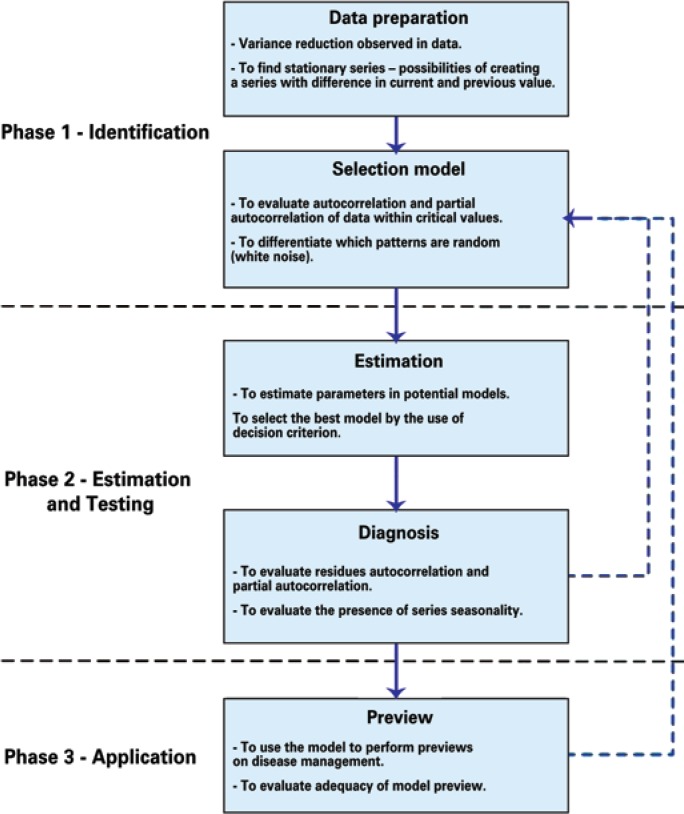
Scheme for the use of Box-Jenkins methodology^(15)^

Figure 1 shows a schematic diagram of the ARIMA model of process estimation. A wide variety of ARIMA models are found. The general format of the nonseasonal model is the AMIRA (p, d, q) being AR: (p=degree of the autoregressive part); I: (d=degree of the first difference involved), and MA: (q= degree of the mean part that is mobile).

The use of the Box-Jenkins methodology (ARIMA model) can be done in three phases^(15)^: identification, estimation and testing, and application. Below, we describe in detail what must be observed in each phase of the process.

### Identification phase

The autocorrelation function (ACF) is a standard tool used to explore time series. This tool enables the user to identify seasonality, cycles, and other patterns in a series. ACF also enables the researcher to identify information concerning a prior period associated with the sequential observation^(15)^.

A stationary series has a “white noise” when mistakes consist of a sequence of uncorrelated random variables. One can understand white noise in errors as the inexistence of patterns, which is equivalent to establishing that mistakes are not correlated. The partial ACF (PACF) is used to measure the associative degree between an observation ( ) and an observation made in two periods before ( ) removing the intermediate period ( )^(15)^. PACF enables the evaluation of the correctness degree of current variables with its previous values, whereas other constant values are kept.

### Graphical data representation

In this first stage, identification of discrepant or less usual data in the series is performed. Transformation of data could be needed also to stabilize the variance reaching the stationary stage.

Stationary data are considered throughout time, along with ACF and PACF. If a time diagram shows that data are dispersed horizontally surrounding a constant mean, ACF and PACF values decrease close to zero rather quickly. If this decrease is not seen, the stationary phase has not occurred yet.

The nonstationary stage could be solved by differentiation. This stage must be evaluated if data are seasonal or not. In the case of seasonal data, the first difference must be obtained from the data. In general, one or two differences are required to transform the data in a stationary series^(15)^. It is important to mention that data in healthcare have a relative variability, and it is difficult to identify these patterns. A way to overcome this limitation is to evaluate the autocorrelation (ie, to evaluate how an observation is related to the prior observation). To convert these data to the stationary stage, the investigator creates a new series of data, based on the differences of the current period in relationship to the previous one. Series are considered stationary when autocorrelation does not show statistically significant results.

Once the stationary stage is reached, the autocorrelation must be retested to verify the possible presence of any residual pattern.

### Phase of estimation and test

After identifying the model, AR and MA parameters, seasonal and nonseasonal, must be determined. In this stage, the traditional method of least squares may be used. A form often used is the maximal likelihood. This form could be understood as a viability measure to check the current sample observations given a particular set of parameter values. Maximal likelihood method enables the investigator to find the values of maximal parameters.

Some parameters can present no statistically significant values (p≥0.05); in such cases, these parameters could be taken away from the study in order to improve the arrangement of data.

However, more than one ARIMA model could work for a data series. A selection criterion is the model that has the least sum of squared errors, although this approach is limited because the sum of squares could decrease and the likelihood could increase only by the input of more data.

Analysis of residues is performed, and the ACF model must show the nonexistence of a significant autocorrelation or a partial autocorrelation between residues. The Portmanteau test could be applied as a complementary means to evaluate the adjustment; a positive test might indicate an inadequate model^(15)^.

If a significant autocorrelation is found, the process of identification must be performed to assess other patterns that yet exist. This comparison could be done with other estimation and prediction techniques using measures of mean error, mean absolute error, square mean error, or Theil's U statistics.

### Phase of enforcement

Predictions with the use of time series analysis should not exceed the first 12 months of the program^(2)^. As mentioned, the first periods of the program could not present a significant impact on the patient. In later periods, significant impact levels could be found. After identification of these result levels, specific goals can be attributed for each period. In longer studies, attention should be given to external factors of the model that may create peaks in time follow-up. Some examples are other technological innovations that reduce disease time or outbreaks of epidemics that increase its effect.

In cases of epidemics, the series could be nonstationary and nonlinear, going from one status to another in a complex manner. In addition, periodic structures of infectious disease epidemics change with time. Therefore, in such cases, short periods of time segments are encouraged to analyze the effects of each segment^(16)^.

## CONCLUSION

Several methods and approaches could be used in the healthcare arena. Time series is an analytical tool to study diseases and resources management at healthcare institutions. The flexibility to follow up and recognize data patterns and provide explanations must not be neglected in studies of healthcare interventions. In this study, the ARIMA model was introduced without the use of mathematical details or other extensions to the model. The investigator or the healthcare organization involved in disease management programs could have great advantages when using analytical methodology in several areas, with the ability to perform provisions in many cases. Despite the analytical possibility by statistical means, this approach does not replace investigators' common sense and experience in disease interventions.
